# *APOE* genetics influence murine gut microbiome

**DOI:** 10.1038/s41598-022-05763-1

**Published:** 2022-02-03

**Authors:** Diana J. Zajac, Stefan J. Green, Lance A. Johnson, Steven Estus

**Affiliations:** 1 Department of Physiology and Sanders-Brown Center on Aging, University of Kentucky, Lexington, KY USA; 2 Genome Research Core, Research Resources Center, University of Illinois at Chicago, Chicago, IL USA; 3 Department of Physiology, University of Kentucky, 789 S. Limestone, Rm. 537, Lexington, KY 40536 USA; 4 Present Address: Genomics and Microbiome Core Facility, Rush University Medical Center, Chicago, IL USA

**Keywords:** Genetic association study, Microbiome

## Abstract

Apolipoprotein E (*APOE*) alleles impact pathogenesis and risk for multiple human diseases, making them primary targets for disease treatment and prevention. Previously, we and others reported an association between *APOE* alleles and the gut microbiome. Here, we evaluated effects of *APOE* heterozygosity and tested whether these overall results extended to mice maintained under ideal conditions for microbiome analyses. To model human *APOE* alleles, this study used *APOE* targeted replacement (TR) mice on a C57Bl/6 background. To minimize genetic drift, homozygous *APOE3* mice were crossed to homozygous *APOE2* or homozygous *APOE4* mice prior to the study, and the resulting heterozygous progeny crossed further to generate the study mice. To maximize environmental homogeneity, mice with mixed genotypes were housed together and used bedding from the cages was mixed and added back as a portion of new bedding. Fecal samples were obtained from mice at 3-, 5- and 7-months of age, and microbiota analyzed by 16S ribosomal RNA gene amplicon sequencing. Linear discriminant analysis of effect size (LefSe) identified taxa associated with *APOE* status, depicted as cladograms to show phylogenetic relatedness. The influence of *APOE* status was tested on alpha-diversity (Shannon H index) and beta-diversity (principal coordinate analyses and PERMANOVA). Individual taxa associated with *APOE* status were identified by classical univariate analysis. Whether findings in the *APOE* mice were replicated in humans was evaluated by using published microbiome genome wide association data. Cladograms revealed robust differences with *APOE* in male mice and limited differences in female mice. The richness and evenness (alpha-diversity) and microbial community composition (beta-diversity) of the fecal microbiome was robustly associated with *APOE* status in male but not female mice. Classical univariate analysis revealed individual taxa that were significantly increased or decreased with *APOE,* illustrating a stepwise *APOE2-APOE3–APOE4* pattern of association with heterozygous animals trending as intermediate in the stepwise pattern. The relative abundance of bacteria from the class Clostridia, order Clostridiales, family Ruminococacceae and related genera increased with *APOE2* status. The relative abundance of Erysipelotrichia increased with *APOE4* status, a finding that extended to humans. In this study, wherein mice were maintained in an ideal fashion for microbiome studies, gut microbiome profiles were strongly and significantly associated with *APOE* status in male *APOE-*TR mice. Erysipelotrichia are increased with *APOE4* in both mice and humans. *APOE* allelic effects appeared generally intermediate in heterozygous animals. Further evaluation of these findings in humans, as well as studies evaluating the impact of the *APOE*-associated microbiota on disease-relevant phenotypes, will be necessary to determine if alterations in the gut microbiome represent a novel mechanism whereby *APOE* alleles impact disease.

## Introduction

Apolipoprotein E (*APOE)* alleles impact multiple facets of the human condition, ranging from Alzheimer’s disease (AD) to cardiovascular disease, metabolic syndrome, obesity, fertility and longevity (reviewed in Ref.^1^). The three primary *APOE* alleles include *APOE3*, which has a 78% minor allele frequency, as well as *APOE4* and *APOE2*, with minor allele frequencies of 14 and 8%, respectively. Regarding AD, *APOE2* reduces AD risk while *APOE4* strongly increases AD risk, both relative to *APOE3* (reviewed in Ref.^2^). This association has prompted intense evaluation of possible mechanism(s) underlying *APOE* effects in AD, resulting in *APOE* allelic association with amyloid-beta (Aβ) clearance, Aβ aggregation and astrocyte stress^3–8^. In the periphery, *APOE2* is associated with decreased low-density lipoprotein (LDL) cholesterol, whereas *APOE4* is associated with increased LDL cholesterol, relative to *APOE3*. While this may account for *APOE* association with cardiovascular disease, the mechanisms underlying *APOE* allelic effects on glucose metabolism, inflammation and innate immunity are less clear^2^. The effects of *APOE* alleles in heterozygous individuals are generally intermediate for phenotypes ranging from cholesterol to AD^1,2^. Elucidating these differential actions of *APOE* alleles may provide insights to these processes.

Several studies have suggested a relationship between *APOE* status, the gut microbiome and AD neuropathology. First, *APOE*-deficient mice display microbiome differences relative to wild-type mice^9^. Second, *APOE4*-targeted replacement (TR) mice were more resistant to gastrointestinal *Cryptosporidium* infection than *APOE3* mice^10^. Third, the *APOE4* allele in humans was associated with better defense against childhood diarrheal diseases in lower income countries^11–13^. Fourth, we and others have recently reported microbiome differences in a comparison of *APOE3* and *APOE4*-TR mice^14–16^. Lastly, several reports have found that Aβ-burden in murine models is reduced in gnotobiotic mice or mice treated with antibiotics^17–20^. The mechanism(s) whereby *APOE* alleles influence the gut microbiome are unclear, although *APOE4* has been associated with a greater inflammatory response to lipopolysaccharide (LPS), a microbiome product common to all gram-negative bacteria, in both humans and mice^21,22^.

To begin to evaluate whether *APOE* allelic effects are dominant, co-dominant, or recessive, we compared animals heterozygous and homozygous for *APOE* alleles. Additionally, we improved our study design for rigor and reproducibility by backcrossing the *APOE*-TR mice to obviate possible genetic drift, maintaining mice with mixed genotypes in the same cages to minimize possible cage effects, and mixing used bedding between cages to ensure a homogenous microbial environment among cages.

## Materials and methods

### Mice

*APOE*3-TR^23,24^ male mice were crossed to *APOE4* and *APOE2* female mice to produce *APOE2/E3* and *APOE3/E4* heterozygous offspring. These mice were then crossed to generate 76 experimental mice that included A*POE2/E2* (N = 6), *APOE2/E3* (N = 12), *APOE3/E3* (N = 5), *APOE3/E4* (N = 8), and *APOE4/E4* (N = 4) female mice and *APOE2/E2* (N = 5), *APOE2/E3* (N = 7), *APOE3/E3* (N = 13), *APOE3/E4* (N = 11), and *APOE4/E4* (N = 5) male mice. Genotypes were determined by TaqMan SNP assays (Thermo). At weaning, mice were separated by sex and housed as mixed genotypes, 2–5 mice per cage (average of 3.7 ± 1.4 (mean ± SD)). Mice were maintained on Teklad Global 18% Protein Rodent Diet. To minimize potential confounding effects of coprophagy (mice feeding partially on cage-mate feces)^25^, approximately 20% of the new bedding was a mixture of used bedding from all the cages. Feces were obtained from this cohort of mice at 3-, 5- and 7-months of age. To obtain feces, mice were temporarily removed from their cage and placed into a clean Styrofoam cup. Fresh fecal pellets were stored at − 80 °C until DNA isolation. All methods were approved by University of Kentucky Institutional Animal Care and Use Committee. This study was carried out in compliance with ARRIVE guidelines.

### Microbiome analysis

Fecal DNA was isolated by using a QIAamp PowerFecal Pro DNA Kit (QIAGEN). Genomic DNA was polymerase chain reaction (PCR) amplified with primers CS1_515F and CS2_806R (modified from the primer set employed by the Earth Microbiome Project (EMP; GTGYCAGCMGCCGCGGTAA and GGACTACNVGGGTWTCTAAT) targeting the V4 regions of microbial small subunit ribosomal RNA genes were generated using a two-stage PCR amplification protocol as described previously^26^. The primers contained 5′ common sequence tags (known as common sequence 1 and 2, CS1 and CS2). First stage PCR amplifications were performed in 10 µl reactions in 96-well plates, using MyTaq HS 2× mastermix (Bioline). PCR conditions were 95 °C for 5 min, followed by 28 cycles of 95 °C for 30′′, 55 °C for 45′′ and 72 °C for 60′′.

Subsequently, a second PCR amplification was performed in 10 µl reactions in 96-well plates. A mastermix for the entire plate was made using MyTaq HS 2× mastermix. Each well received a separate primer pair with a unique 10-base barcode, obtained from the Access Array Barcode Library for Illumina (Fluidigm, South San Francisco, CA; Item# 100-4876). Cycling conditions were: 95 °C for 5 min, followed by 8 cycles of 95 °C for 30′′, 60 °C for 30′′ and 72 °C for 30′′. Samples were then pooled, purified, and sequenced on an Illumina MiniSeq platform employing paired-end 2 × 153 base reads. Fluidigm sequencing primers, targeting the CS1 and CS2 linker regions, were used to initiate sequencing. De-multiplexing of reads was performed on instrument. Library preparation, pooling, and sequencing were performed at the University of Illinois at Chicago Genome Research Core (GRC) within the Research Resources Center (RRC).

Forward and reverse reads were merged using PEAR^27^ and trimmed based on a quality threshold of *p* = 0.01. Ambiguous nucleotides and primer sequences were removed and sequences shorter than 225 bp were discarded. Chimeric sequences were identified and removed using the USEARCH algorithm with a comparison to the Silva 132_16S reference database^28,29^. Amplicon sequence variants (ASVs) were identified using DADA2^30^ and their taxonomic annotations determined using the UCLUST algorithm and Silva 132_16S reference with a minimum similarity threshold of 90%^28,29^. Sequence processing and annotation was performed by the Research Informatics Core (RIC) within the RRC.

This sequencing effort yielded 10,162,042 reads. Raw sequence data files were submitted in the Sequence Read Archive (SRA) of the National Center for Biotechnology Information (NCBI) under the BioProject Identifier PRJNA787634. Two samples with fewer than 30,000 reads each were discarded. Since *APOE* effects may be sex dependent^16^, microbiomes from male and female mice were analyzed separately. Average read counts per sample for the 3-month males was 48,725, 3-month females was 49,499, 5-month males was 45,985, 5-month females was 49,939, in 7-month males was 50,975, in 7-month females was 51,931. Using MicrobiomeAnalyst^31^ (updated version February 2021), samples were rarified to the minimum library size, which for 3-month males was 36,421, 3-month females was 37,069, 5-month males to 30,738, 5-month females to 35,467, 7-month males to 35,452, and 7-month females to 36,730. Low abundance ASVs were removed, i.e., ASVs with < 3 counts in > 90% of the samples were removed, and low variance ASVs were also removed, i.e., ASVs whose inter-quantile range was in the lowest 10%^31^. These corrections reduced the number of ASVs from 263 to 63 ASVs in 3-month males and females, 67 in 5-month females, 69 in 5-month males, 68 in 7-month females and 66 in 7-month males. Count data were normalized with a centered log-ratio transformation. Regarding *APOE* genetics, the results were analyzed as separate genotypes or as pooled alleles, i.e., *APOE2/E3* heterozygous mice were grouped with *APOE2/E2* mice while *APOE3/E4* mice were grouped with *APOE4/E4* as described in other *APOE* studies^32,33^. This resulted in 12 *APOE2*, 13 *APOE3* and 16 *APOE4* male mice and 18 *APOE2*, five *APOE3* and 12 *APOE4* female mice.

Bacteria associated with *APOE* were identified by a linear discriminant analysis effect size (LefSe) approach^34^. Significance thresholds were set to 0.05 for the alpha values for Kruskal–Wallis/Wilcoxon tests and 2.0 for the logarithmic linear discriminant analysis (LDA) score, using a one-against-all multi-class analysis approach. These results were then plotted as a cladogram to document the phylogenetic relatedness of *APOE* allelic associations with the bacteria at each taxonomic level. Results are also presented as an LDA histogram.

Alpha-diversity was assessed using the Shannon H diversity index^35^ with *APOE* statistical significance determined by Kruskal–Wallis tests. Additional alpha-diversity tests included Margalef taxon richness, Pielou’s evenness and the Simpson index with *APOE* statistical significance determined by Jonckheere–Terpstra nonparametric tests. Beta-diversity was assessed using Principal Coordinates Analysis (PCoA) of Bray–Curtis matrices with statistical significance determined by Permutational Multivariate Analysis of Variance (PERMANOVA)^36^. Taxonomic levels that associate with *APOE* status were determined using a classical univariate analysis with a Kruskal–Wallis test. A false discovery rate (FDR) approach was used to correct for multiple testing^31^. Heatmaps of family-level bacterial relative abundances were generated for male and female mice as a function of *APOE* status by using the Ward analysis of variance clustering algorithm, which is based on Pearson Correlation Coefficient distance measures.

### Ethics approval

Animal studies were performed in compliance with the Institutional Animal Care and Use Committee at University of Kentucky.

### Consent for publication

Each of the authors have reviewed the manuscript and approved it for publication.

## Results

**Figure 1:**
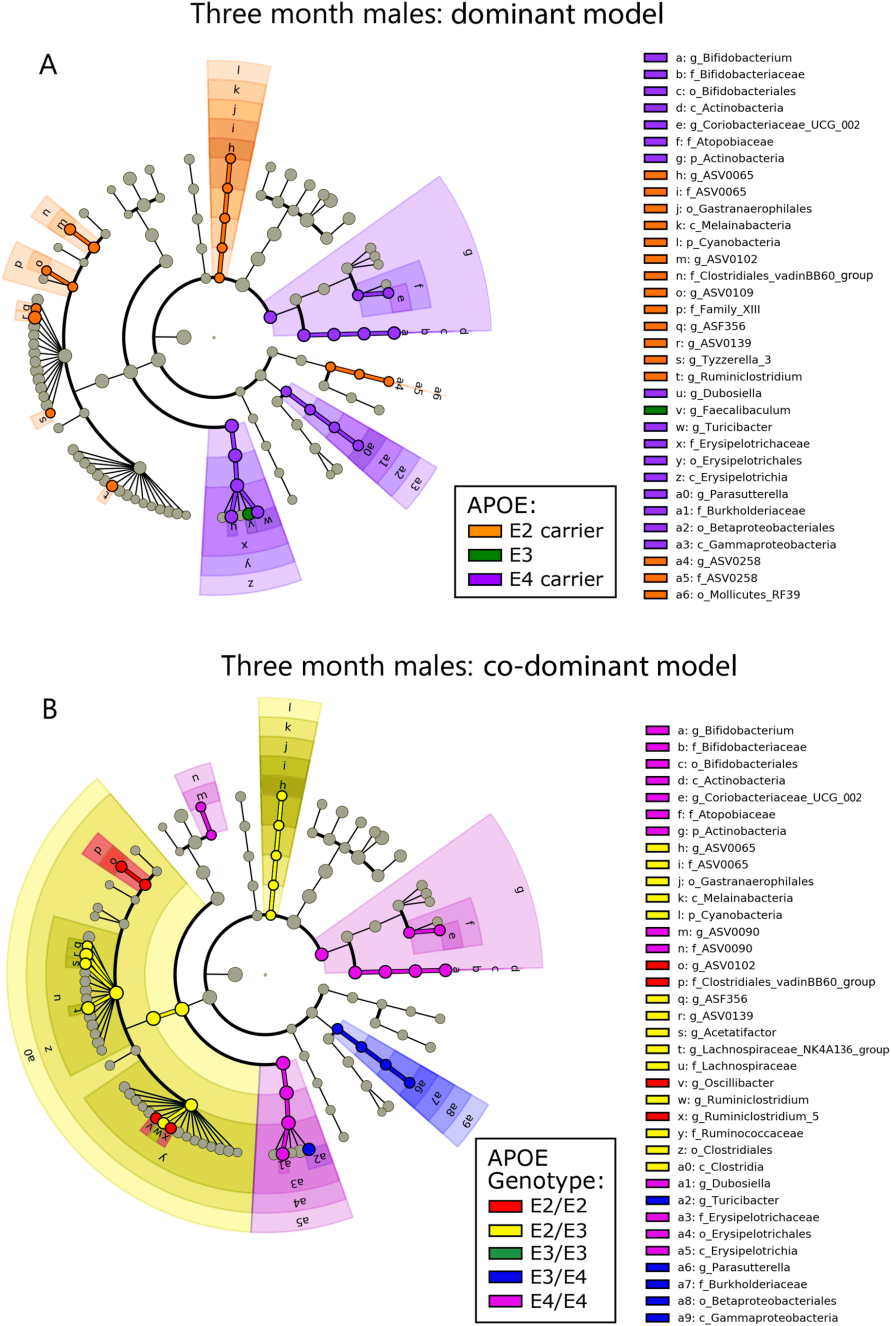
Cladograms reveal microbial phylogenetic branches associated with *APOE* status in males. Taxa are represented as nodes and are connected by lines based on the phylogenetic relatedness of all taxa present in each experimental cohort. For example, the end node, a. represents the genus *Bifodobacterium* which is connected to other nodes representing higher level taxa related to *Bifodobacterium* including; b. the family Bifidobacteriaceae, c. the order Bifidobacteriales, and d. the class Actinobacteria. Many taxa are associated with *APOE* genetics, with node colors indicating the *APOE* associated with highest levels of that taxa. Statistical significance reflects both p < 0.05 for Kruskal–Wallis tests and a logarithmic LDA score > 2.0.

**Figure 2:**
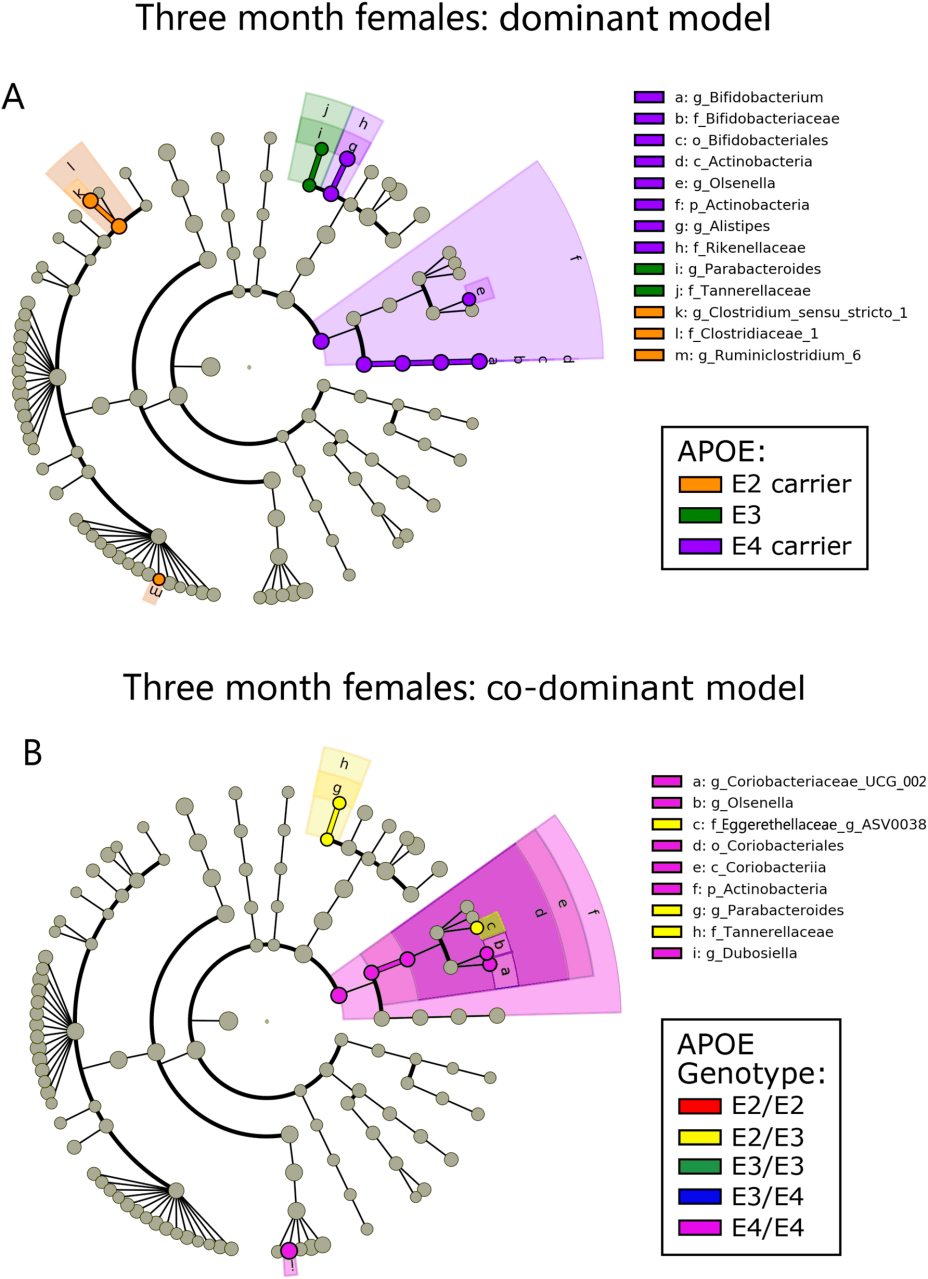
Cladograms reveal microbial phylogenetic branches associated with *APOE* status in females. Taxa significantly associated with *APOE* are highlighted (p < 0.05 for Kruskal–Wallis tests and a logarithmic LDA score > 2.0).

To investigate the hypothesis that *APOE* is associated with gut microbial community structure, we began with LefSe analysis and visualized the results with cladograms (Figs. 1, 2, Figs. S1, S2). This robust approach provides a visual means to identify statistically significant and phylogenetically-related taxa associated with *APOE* status^34^. These LefSe results are also presented as LDA histograms to provide a quantitative representation of the LefSe analyses (Fig. S3). Results are presented with *APOE* status stratified as *APOE2* carriers, *APOE3* and *APOE4* carriers (pooled) (Figs. 1A, 2A) and with *APOE* status as separate genotypes (Figs. 1B, 2B). These two representations of the data provide insights into whether *APOE* allelic effects are dominant or co-dominant. Robust gut microbiome differences were observed in male mice compared to female mice at 3-months of age (Figs. 1, 2) with similar results found at 5- and 7-months of age (Figs. S1, S2). The microbiome of both male and female mice showed *APOE4*-associated increases in members of the Actinobacteria phylum (Figs. 1, 2). In contrast, only male mice showed *APOE4*-associated increases in the Erysipleotrichia and Gammaproteobacteria classes and *APOE2*-associated increases in the Cyanobacteria phylum. To gain further insights into these findings, we parsed the results into individual genotypes. This expanded our findings by showing that members of the Clostridia class were significantly associated with *APOE2/E2* and *APOE2/E3* (Fig. 1B). Overall, these results indicated that a subset of bacteria were consistently associated with *APOE* status, especially in males. The male population captured the majority of taxa significantly associated with *APOE* in the female population. In the following results, we present analyses of data from male mice at 3-months of age with analyses for all ages and sexes included within the Supplemental Files.

**Figure 3:**
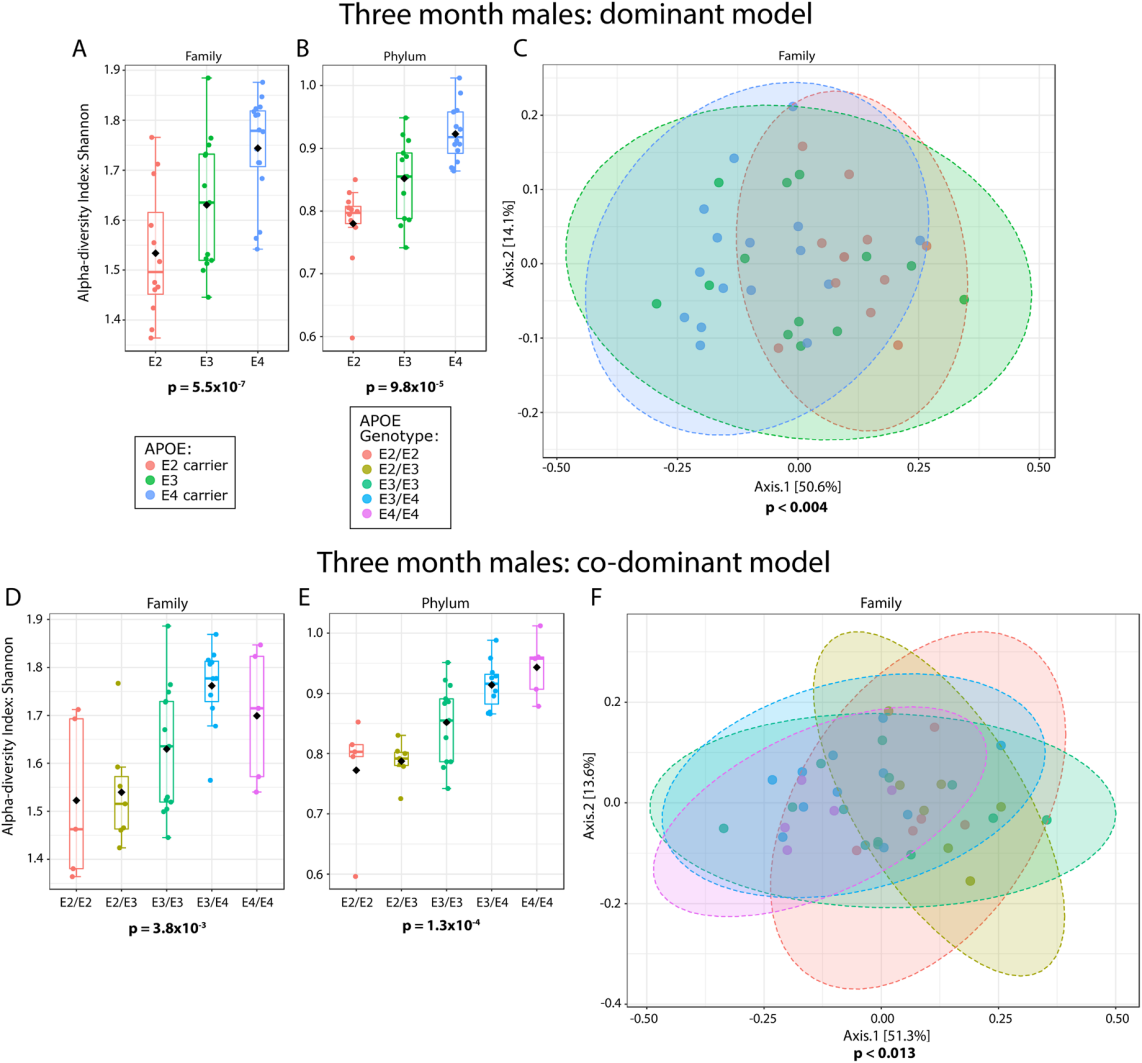
Microbiome alpha- and beta-diversity as a function of *APOE*. Alpha-diversity is depicted as boxplots (**A,B,D,E**) and beta-diversity as PCoA plots (**C,F**). These results are from male mice at 3-months of age. Statistical significance for the findings is indicated below each graph. Ellipses in C and F represent 95% confidence intervals. Dominant model (C) R^2^ = 0.171 and co-dominant model (F) R^2^ = 0.206. Beta-diversity was also analyzed using a PERMDISP, which had no significant p-values, indicating that variances were not significantly different as a function of *APOE*.

Microbiome alpha- (within sample) diversity was assessed by the Shannon H index and Simpson index, measures of taxon richness and evenness, as well the Margalef taxon richness index and evenness index^37^. A robust association between alpha-diversity and *APOE* was not detected in female mice (Shannon index p-values in Table 1, all measures of alpha-diversity in Supplemental Files). In contrast, male mice showed a stepwise trend towards higher alpha-diversity with *APOE2-APOE3-APOE4* at the genus level, and this trend became more defined and statistically significant at every higher level through phylum (phylum and family depicted in Fig. 3, all levels presented in Table 1 and Supplemental Files, additional ages, and female data in Table S1). When the results are parsed into separate genotypes, the alpha-diversity of the *APOE* heterozygous animals tended to be intermediate relative to the homozygous animals. Hence, *APOE* was associated with alpha-diversity in male but not female mice and *APOE* allelic affects appeared co-dominant.

**Table 1:** Microbiome alpha-diversity was significantly associated with *APOE* status in 3-month male but not female mice.

Alpha-diversity (Shannon index)		
Taxanomic level	3 month p-values	
	Males dominant	Males co-dominant
Feature	4.01E−01	1.02E−01
Genus	4.01E−01	1.02E−01
Family	**5.49E**−**04**	**3.81E**−**03**
Order	**7.68E**−**05**	**5.05E**−**04**
Class	**7.68E**−**05**	**5.05E**−**04**
Phylum	**9.77E**−**05**	**1.28E**−**04**

P-values reflect nominal p-values and were determined using Kruskal–Wallis tests. Values for 5- and 7-month male mice and female mice are presented in Tables S1 and S2. Raw data for alpha-diversity indices are provided in Supplemental Files.

Significance p-values are in bold font.

Beta-diversity is a measure of between sample microbial communities based on their composition. Beta-diversity was visualized by using PCoA based on Bray–Curtis distance matrices^38–41^, and analyzed using a PERMANOVA. We found that *APOE* status was significantly associated with microbiome beta-diversity in male mice (Fig. 3, Table 2, additional ages and female in Table S2). When the data were analyzed with each separate *APOE* genotype, beta-diversity was still significant at each of the same taxonomic levels. Overall, these results demonstrate that the microbiome is robustly associated with *APOE* genetics in male mice.

**Table 2:** Microbiome beta-diversity significantly associated with *APOE* status in male, but not female, mice.

Beta-diversity (PERMANOVA)			
Taxanomic level		3 month	
		Males -dominant	Males co-dominant
Feature	p-value	** < 0.002**	** < 0.004**
	R^2^	0.14	**0.20**
Genus	p-value	** < 0.001**	** < 0.008**
	R^2^	0.15	0.18
Family	p-value	** < 0.004**	** < 0.013**
	R^2^	0.17	0.21
Order	p-value	** < 0.004**	** < 0.015**
	R^2^	0.21	0.24
Class	p-value	** < 0.004**	** < 0.015**
	R^2^	0.21	0.24
Phylum	p-value	** < 0.035**	< 0.112
	R^2^	0.12	0.15

The R^2^ values represent the proportion of the variance captured by *APOE* alleles. The PERMANOVA results were derived from 999 permutations. Values for 5- and 7-month male mice and female mice are contained within Tables S3 and S4.

Significance p-values are in bold font.

**Figure 4:**
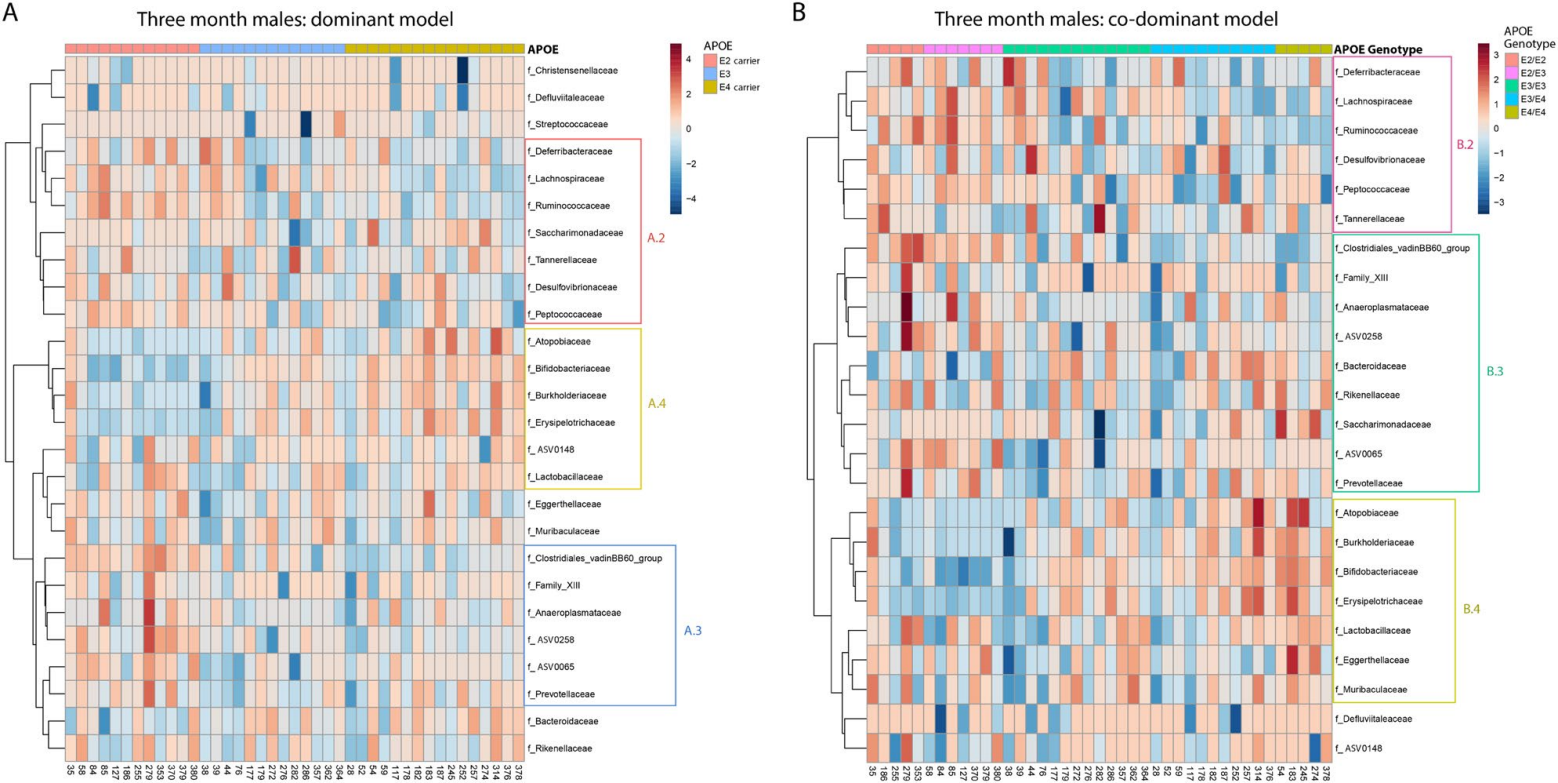
Heatmaps depict overall microbiota profiles grouped by *APOE* status. These heatmaps depict per-sample relative abundance for family-level bacteria in female and male mice. Columns were grouped by *APOE* status, rows were grouped by the Ward clustering algorithm using Pearson Correlation Coefficient distance measures. Colored boxes highlight groups of taxa that follow either an *APOE2*- (A.2 and B.2), *APOE3*- (A.3 and B.3) or *APOE4*-associated pattern (A.4 and B.4).

Variation in bacterial relative abundance per sample was visualized using heatmaps, as seen in Fig. 4. While cladograms identify bacterial phylogenetic branches that correlate with high abundance in association with a specific *APOE* status, heatmaps provide a per sample depth of information for each *APOE* status. Inspection of the heatmaps suggests that the data are relatively complex although some patterns of taxa association with *APOE* status are discernible, e.g., Ruminococcaceae and Erysipelotrichaceae in male mice (Fig. 4).

**Figure 5:**
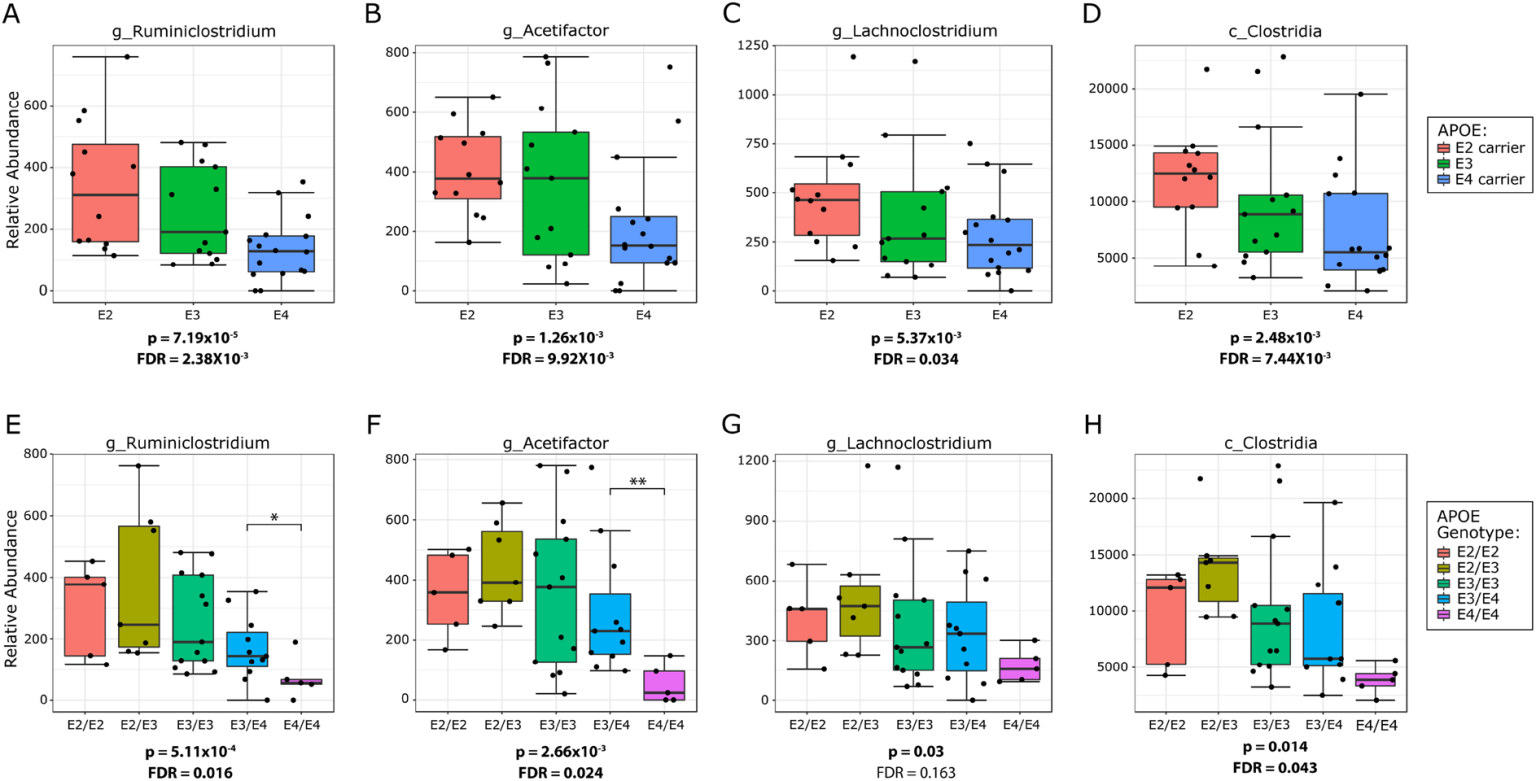
The phylogenetic branch defined by Clostridia and its lower taxa shows a significant association with *APOE* in male mice. The relative abundance of each depicted bacteria was significantly associated with *APOE* status. The relative abundance of these bacteria decreased in a stepwise fashion from *APOE2* to *APOE3* to *APOE4*. P values have been corrected using an FDR approach. (**A–D**) Are the plots depicted using the dominant model representation, while (**E–H**) are the plots depicted using the co-dominant model representation. These data are derived from the 3-month male mice with data for all ages provided in Supplementary Tables S5.0–SS7.5.

Although the use of LefSe analysis and cladograms provides insights into the microbiome and have been optimized for this purpose^34^, another perspective is provided by taxon-by-taxon classical univariate analysis using a Kruskal–Wallis test for significance and an FDR correction for multiple testing. To highlight the taxa most robustly associated with *APOE*, we applied classical univariate analysis to identify results that were significant with both approaches. Classical univariate analysis of the 3-month old female mice found no ASVs, genera, families, orders, classes, or phyla that were significantly associated with *APOE* in either *APOE* model. However, this approach applied to 3-month old male mice in the dominant model found 12 *APOE*-associated ASVs, as well as 12 genera, six families, five orders, five classes, and one phylum (statistics for all taxa in each age group are listed in Supplementary Tables S1.1–S3.4). In the co-dominant model, this approach found eight ASVs, eight genera, three families, four orders, four classes, and no phyla that were significantly associated with *APOE*. A graphical representation of the findings in 3-month old male mice is depicted in Fig. 5. Several bacteria showed stepwise associations with *APOE* on multiple taxonomic levels and were overall increased with *APOE2*. For example, the Clostridia class, Clostridiales order and two major families within this phylogenic branch, Ruminococcaceae and Lachnospiraceae showed an increase in their relative abundance from *APOE4* to *APOE3* to *APOE2* (Fig. 5)*.* The most abundant genera within the Ruminococcaceae family significantly associated with *APOE* were *Ruminiclostridium* (Fig. 5A,E)*, Ruminiclostridium_5* and *Ruminiclostridium_9,* which in aggregate represent approximately half of the Ruminococcaceae family*.* At 5- and 7-months of age, other genera within Ruminococcaceae were associated with *APOE* (Supplemental Tables S5.0–S7.5). Genera within the other major family, Lachnospiraceae*,* that increased with *APOE2* were *Acetifactor* and *Lachnoclostridium* (Fig. 5B,C,F,G)*.*

**Figure 6:**
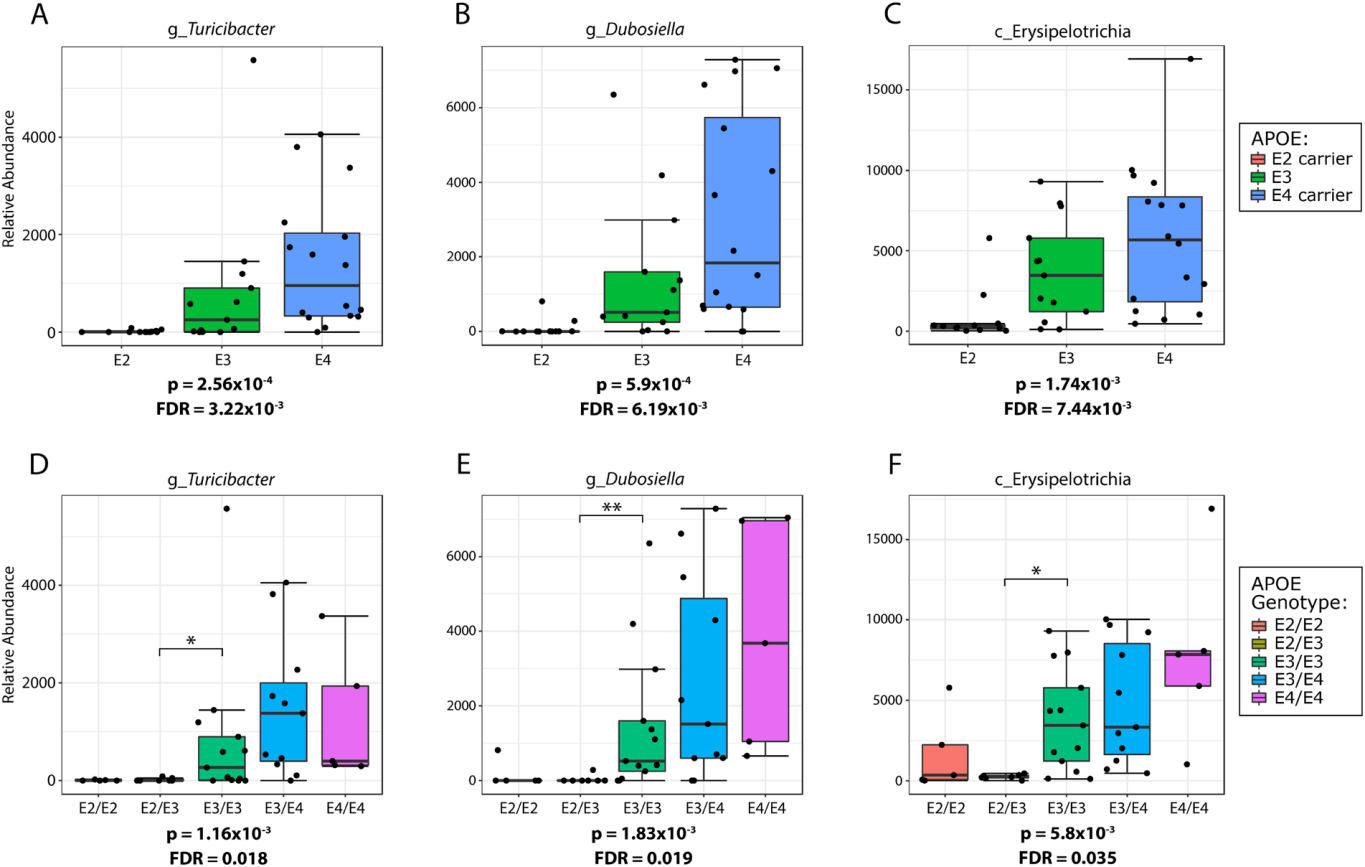
The phylogenetic branch defined by Erysipelotrichia and its lower taxa shows a significant association with *APOE* in male mice. All depicted bacteria were significantly associated with *APOE* status. The relative abundance of these bacteria increased in a stepwise fashion from *APOE2* to *APOE3* to *APOE4.* P values have been corrected using an FDR approach. (**A–C**) Are the plots depicted using the dominant model representation, while (**D–F**) are the plots depicted using the co-dominant model representation. These results are derived from 3-month male mice.

In contrast, the relative abundance of other taxa was increased in *APOE4* mice, most notably bacteria within the class Erysipelotrichia class, confirming the findings from the LefSe cladograms (Fig. 1). Bacteria within the Erysipelotrichia branch that were significantly associated with *APOE* included the order Erysipelotrichiales, its family Erysipelotrichaceae and its genera *Turicibacter* and *Dubosiella* (Fig. 6, data for all ages shown in Supplementary Tables S5.0–S7.5). Consistent within this branch, bacterial relative abundance was near zero in the *APOE2* mice, moderate in *APOE3* and highly enriched in *APOE4* (Fig. 6). Hence, both the LefSe and classical approaches identified members of the Clostridia class as enriched in *APOE2* mice while members of the Erysipelotrichia class were enriched in *APOE4* mice.

To discern whether the Clostridiales and Erysipelotrichiales phylogenetic branches associated with *APOE* in this murine *APOE*-TR model are also associated with *APOE* in humans, we turned to a recent genome wide association study (GWAS) that evaluated the relationship between the gut microbiome and human polymorphisms^42^. This meta-analysis included data from as many as 18,340 individuals^42^. The only genetic locus that reached genome wide statistical significance was rs182549, which is associated with lactose intolerance. Interestingly, this SNP is modestly associated with the risk of Alzheimer’s disease (p = 0.003, N = 445,779)^43^, consistent with the possibility that the gut microbiome may influence AD risk. Focusing on *APOE*, the alleles of *APOE2*, *APOE3* and *APOE4* are defined by two SNPs, rs7412 and rs429358. The minor allele of rs7412 defines *APOE2* while the minor allele of rs429358 determines *APOE4* status. The Clostridiales and Erysipelotrichiales phylogenetic branches were not significantly associated with rs7412 (*APOE2*) at any phylogenetic level. However, the class Erysipelotrichia, the order Erysipelotrichales and the family Erysipelotrichaceae were nominally associated with rs429358 (Table 3). For each of these taxa, the minor *APOE4* allele was associated with an increase in the relative abundance of these bacteria, reproducing the findings observed in the murine *APOE*-TR model.

**Table 3:** Bacteria in the Erysipelotrichia phylogenetic branch are nominally associated with rs429358 in humans.

Bacteria	SNP	Reference Allele	Effect Allele	Beta	SE	p value	N
Class: Erysipelotrichia	rs429358	T	C	0.032	0.015	**0.035**	18,097
Order: Erysipelotrichales	rs429358	T	C	0.032	0.015	**0.035**	18,097
Family: Erysipelotrichaceae	rs429358	T	C	0.032	0.015	**0.035**	18,097
Genus: Turicibacter	rs429358	T	C	0.001	0.021	0.87	8921

The positive beta values reflect that the bacterial taxa are increased with the minor *APOE4* allele of rs429358. These results combine data from men and women and are supplemental data from a large microbiome genetics study^42^.

Significance p values are in bold font.

## Discussion

The primary finding reported here is that murine gut microbiome profiles are significantly associated with *APOE* status and *APOE* alleles appeared to act in a co-dominant fashion in heterozygous mice. These findings are strengthened given that the *APOE*-TR mice were maintained in an optimized fashion for microbiome analyses. The microbiome association with *APOE* was observed in alpha- and beta-diversity, encompasses multiple bacterial lineages and was predominately observed in male mice. Both LefSe and classical univariate analyses identified specific taxa that were associated with *APOE*. This association occurred in a stepwise fashion in the mice with the progression from *APOE2-APOE3-APOE4*. The stepwise association between indices of the gut microbiome and *APOE2-APOE3-APOE4* reported here are reminiscent of *APOE* allelic association with other phenotypes ranging from LDL-cholesterol to AD risk^1,2^. Additionally, at least one of these associations, an increase in the relative abundance of Erysipelotrichia with *APOE4*, also has been observed in the human gut microbiome^42^. Moreover, the stepwise trends were also seen in the heterozygous mice, which reflected resulting intermediate effects of *APOE* on the gut microbiome. Overall, these findings confirm and extend prior reports that *APOE* genetics are associated with the gut microbiome^14–16^.

To identify the impact of *APOE* alleles on the microbiome, we used several approaches in this study, including alpha-diversity, beta-diversity, LefSe and classical univariate analyses. Alpha- and beta-diversity analyses aggregate multiple variables to provide an assessment of overall microbiome diversity and of microbiome profile similarity, respectively. In contrast, LefSe and classical univariate analyses provide an indication of differences in the relative abundance of specific taxa between experimental groups. These analyses were done by pooling the genotypes in a way representative of a dominant model of allelic effect and as individual genotypes representative of a co-dominant model of allelic effect. In this discussion, we will highlight the primary significant findings from these various analyses.

*APOE4* was associated with increased alpha-diversity as assessed by the Shannon H index. A stepwise progression was observed in the dominant model representation with lowest alpha-diversity in *APOE2*, moderate in *APOE3* and highest in *APOE4.* The co-dominant model representation showed an intermediate effect in the heterozygous animals following the stepwise *APOE* trend. Alpha-diversity is a measure of the number of distinct taxa and the evenness of these numbers across taxa. High alpha-diversity in the gut microbiome has been associated with improved gut health and microbiome homeostasis (reviewed in Ref.^44^). The *APOE4* association with increased alpha-diversity observed here is consistent with prior observations that *APOE4* is associated with better response to diarrheal infections in a third-world environment^12,13^. Indeed, the enrichment of *APOE4* in people indigenous to Amazonian basin has been proposed to be a result of evolutionary selection in this environment with insufficient sanitation^45^.

A primary finding of this study was that both the LefSe and classical univariate analyses found that taxa within the Clostridia class were increased with *APOE2* status, confirming results from our prior study^14^ and that of Tran et al.^15^. This phylogenetic branch included the Clostridiales order, Ruminococcaceae family and several genera within this family. The Clostridiales order was increased in *APOE2* mice compared to *APOE3* and *APOE4* mice, with the heterozygous mice as intermediates. This was most robust in the 3-month males with similar findings at 5- and 7-months. The two major bacterial families within this order, Ruminococcaceae and Lachnospiraceae, were also both increased with *APOE2*. The stepwise fashion of the decline in Ruminnococcaceae relative abundance from *APOE2* to *APOE3* to APOE4 confirms the stepwise pattern seen previously^14,16^ and extends it along the phylogenetic branch from the Clostridia class to associated genera, such as *Ruminiclostridium, Ruminiclostridium_5, Ruminiclostridium_9*. Interestingly, Tran et al. also reported an increase in relative abundance of the Clostridiales order and Ruminococcaceae family in *APOE2/E3* humans compared to *APOE3/E4* and *APOE4/E4* humans^15^. This suggests that this increase in Clostridiales and Ruminococcaceae with *APOE2* may extend to humans. Two additional genera in the Clostridia class, within the Lachnospiraceae family, i.e., *Acetifactor* and *Lachnoclostridium,* also increased with *APOE2* status in the current study. However, this finding was not replicated by Tran et al., who reported that Lachnospiraceae increased in *APOE4* mice compared to *APOE3* mice^15^.

Our study strengthens the associations between *APOE* status and the Clostridiales order, Ruminococcaceae family and related genera, and the *Acetifactor* and *Lachnoclostridium* genera by demonstrating a stepwise pattern with *APOE* allelic status across the entire phylogenetic branch from the Clostridia class down to related genera. Ruminococcaceae and Lachnospiraceae are bacterial families that highly express genes responsible for the metabolism of resistant starches in the large intestine, generating short chain fatty acids (SCFAs). The presence of SCFAs in the gut affect human health in general (reviewed in Refs.^46,47^) and have been reported to promote microglial maturation and function in particular^48^. Treatment with SCFAs has been shown to reduce microglial pro-inflammatory signals and promote a homeostatic profile that is neuroprotective^49–52^. Considering these findings relative to disease pathology associated with the stepwise *APOE2-APOE3-APOE4* phenotype, we propose a tentative model wherein (i) *APOE2* is associated with an increase in the relative abundance of microbiome bacteria Ruminococcaceae*, Acetifactor* and *Lachnoclostridium*, relative to *APOE3* and *APOE4*, (ii) this shift in bacterial profile increases the production of SCFAs and (iii) this increase in SCFAs promotes microglial homeostasis and disease-ameliorating signaling, as suggested by robust genetic evidence^53–61^, (reviewed in Refs.^62,63^). While speculative, this model serves as a framework for future studies.

Another primary finding detected by both the LefSe and classical univariate analyses was that the *Erysipelotrichia* phylogenetic branch was significantly associated with *APOE* status in a stepwise *APOE2-APOE3-APOE4* pattern with heterozygous mice acting as intermediates. This finding appeared to extend to humans and replicates the increase of the Erysipelotrichaceae family in *APOE4* mice that we observed previously^14^. This parallels the association of the Erysipelotrichia class, Erysipelotrichales order and Erysipelotrichaceae family with the *APOE4* minor allele rs429358 in human GWAS data. Our current study also extends this finding from the Erysipelotrichia phylogenetic order to its major genera, i.e., *Turicibacter* and *Dubosiella*. However, Tran et al. reported Erysipelotrichaceae were significantly increased in *APOE3* compared to *APOE4* mice. Erysipelotrichaceae has been shown to increase in animals fed a high-fat diet and to decrease in patients on a low-fat diet^64,65^. Hence, diet variation between the mice in our study and those of Tran et al. may account for the Erysipelotrichaceae difference, noting that our Teklad Global 18% (2018) chow has a fat content that accounts for 18% of total calories, whereas the RPM3, Special Diet Services chow used in the Tran et al. study has a fat content that accounts for 12% of total calories^15^. Since *APOE* genetics have been associated with BMI and obesity^1^, there may be a complex interplay between diet, *APOE* genotype and relative abundance of Erysipelotrichaceae in the gut.

Although the cladograms depict these results as phylogenetic trees, and the relatedness of taxa could imply functional relatedness, a limitation of this study is that findings were not extended to a functionality analysis. A future extension of these findings would be an in-depth analysis of the functional phenotypes of the significantly associated taxa. Future metabolomic studies would provide information regarding differences in the gut as a function of *APOE* genetics.

## Conclusions

In this study in which mice were maintained with optimized conditions for microbiome analysis, we report a significant association between *APOE* status and gut microbiome profiles in 3-month male mice that reproduces at 5- and 7-months of age. The Clostridia class, Clostridiales order, its related family Ruminococcaceae, as well as related genera *Ruminoclostridium,* and *Acetifactor* and *Lachnoclostridium* of the Lachnopsiraceae family, increase with *APOE2,* which may reflect an increase in resistant starch metabolism with *APOE2*, and a possible impact on SCFA levels. The Erysipelotrichia class, Erysipelotrichiales order, Erysipelotrichaceae family, and *Turicibacter* and *Dubosiella* genera increase with *APOE4.* The findings with the Erysipelotrichia phylogenetic branch appear to extend to humans. Both the dominant model representation and the co-dominant model representation of the data showed stepwise *APOE* effects on the gut microbiome, and the comparison of the two provides a more robust understanding of the allelic effects. Understanding the effects of *APOE* genetics on the gut microbiome may provide novel approaches to counter deleterious *APOE* genetic effects on human disease.

## Supplementary Information

Supplementary Information 1.

Supplementary Information 2.

Supplementary Legends.

Supplementary Tables.

Supplementary Figure S1.

Supplementary Figure S2.

Supplementary Figure S3.

## Data Availability

Raw sequence data (FASTQ files) were deposited in the National Center for Biotechnology Information (NCBI) Sequence Read Archive (SRA), under the BioProject identifier PRJNA787634.
