# Supplementary material for: APOE genetics influence murine gut microbiome
Source: Sci Rep. 2022 Feb 3;12:1906. doi: 10.1038/s41598-022-05763-1 (PMC8814305; doi:10.1038/s41598-022-05763-1)
Supplement: Supplementary file 3 — Supplementary Legends. [file 41598_2022_5763_MOESM3_ESM.docx]

**Table S1. Alpha-diversity p-values for all ages and sexes using the dominant model for *APOE* grouping.**

**Table S2. Alpha-diversity p-values for all ages and sexes using the co-dominant model for *APOE* grouping.**

**Table S3. Beta-diversity p-values and R^2^ values for all ages and sexes using the dominant model for *APOE* grouping.**

**Table S4. Beta-diversity p-values and R^2^ values for all ages and sexes using the co-dominant model for *APOE* grouping.**

**Figure S1-S2. Cladograms for five- and 7-month female and male mice, respectively, reveal microbial phylogenetic branches associated with *APOE* status**. These results depict taxa significant associated with *APOE* status by LefSe analysis using the dominant model representation.

**Figure S3.** **LDA histograms for 3-month male mice indicate taxa that significantly associate with *APOE*. A** presents the results using the dominant model representation, while **B** presents the results using the co-dominant model representation. Taxa are significant if they pass the cutoff of 2.
