# Supplementary figures and images for: APOE genetics influence murine gut microbiome
Source: Sci Rep. 2022 Feb 3;12:1906. doi: 10.1038/s41598-022-05763-1 (PMC8814305; doi:10.1038/s41598-022-05763-1)

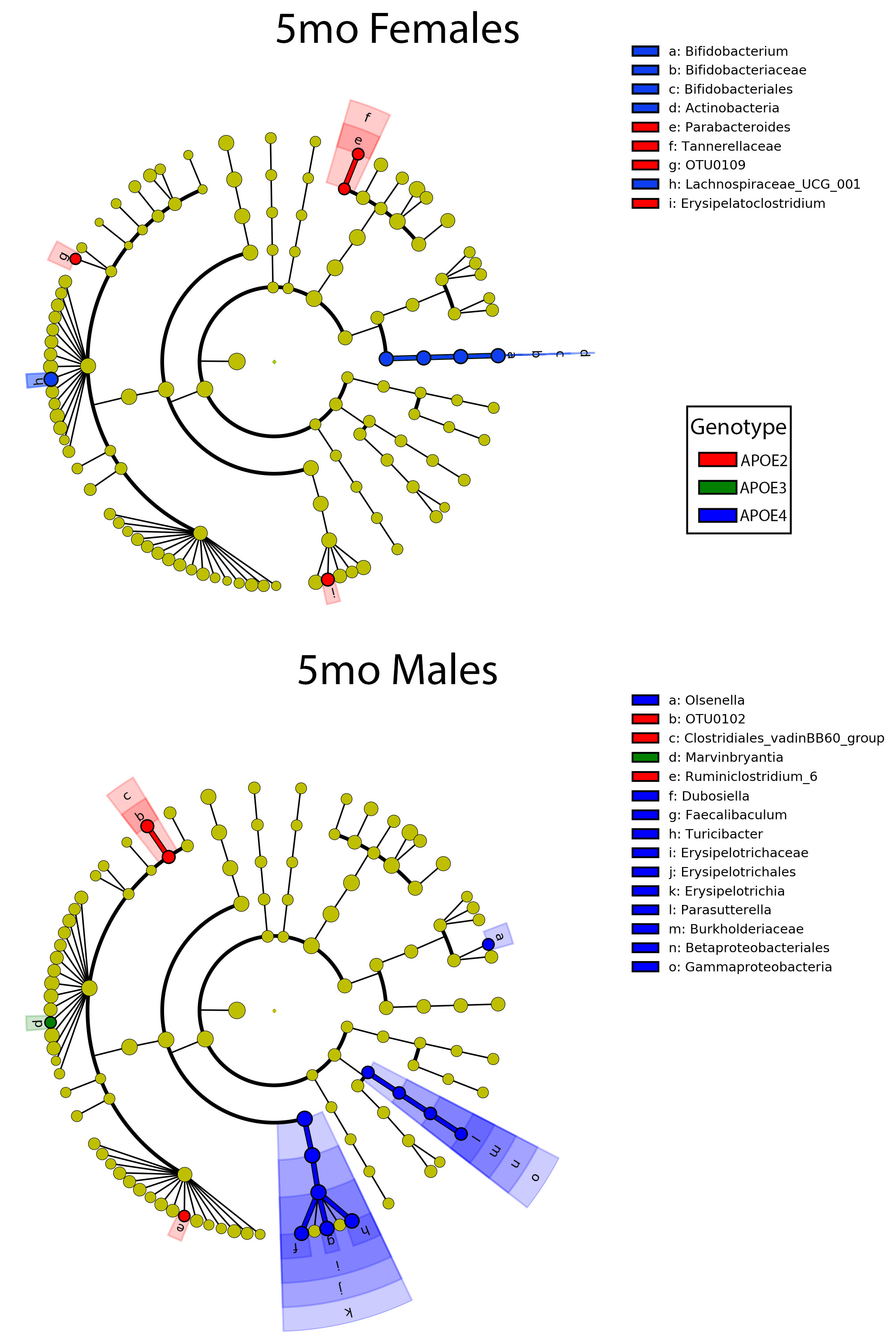

Supplement: Supplementary file 5 — Supplementary Figure S1. [file 41598_2022_5763_MOESM5_ESM.jpg]

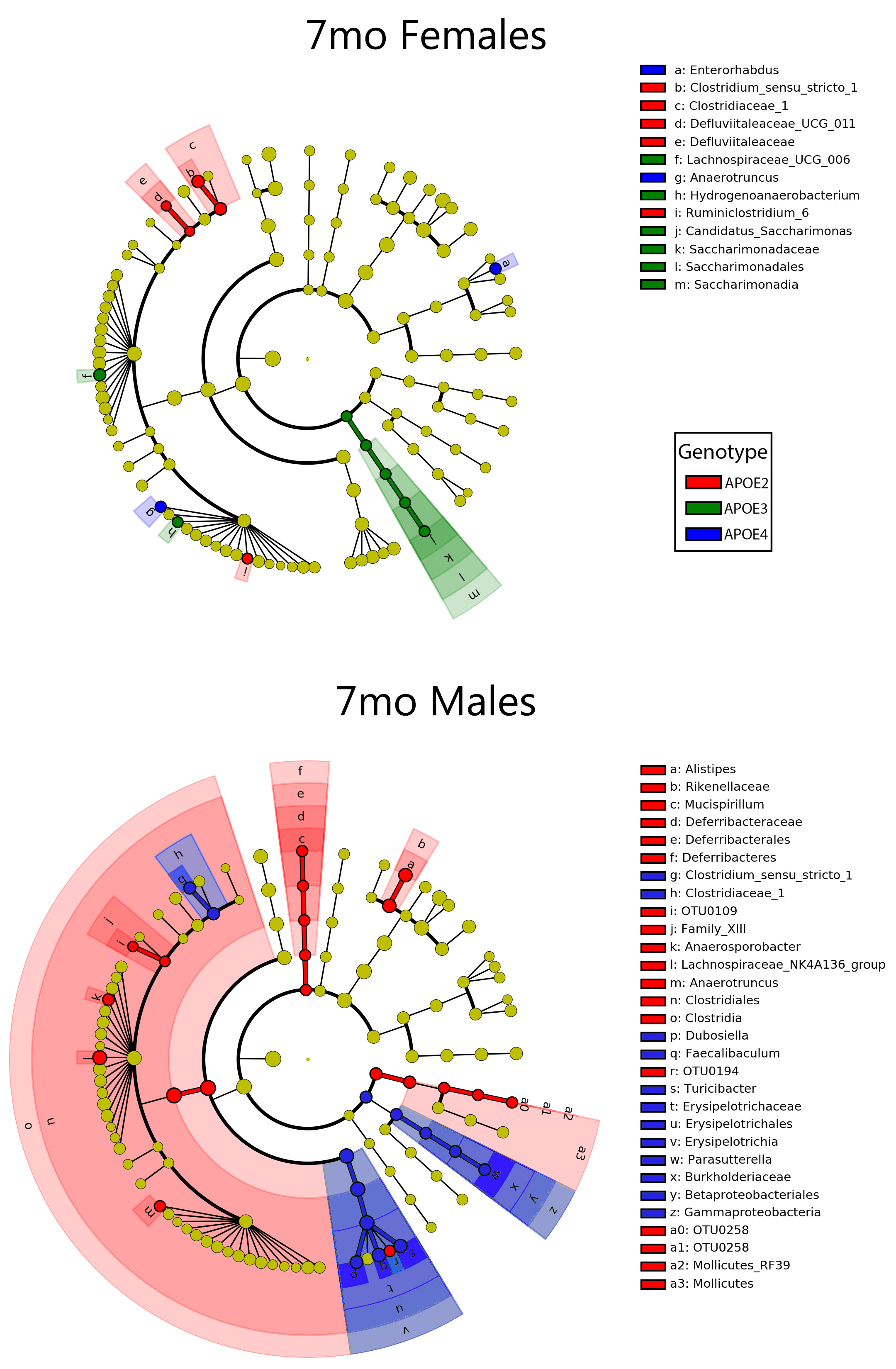

Supplement: Supplementary file 6 — Supplementary Figure S2. [file 41598_2022_5763_MOESM6_ESM.jpg]

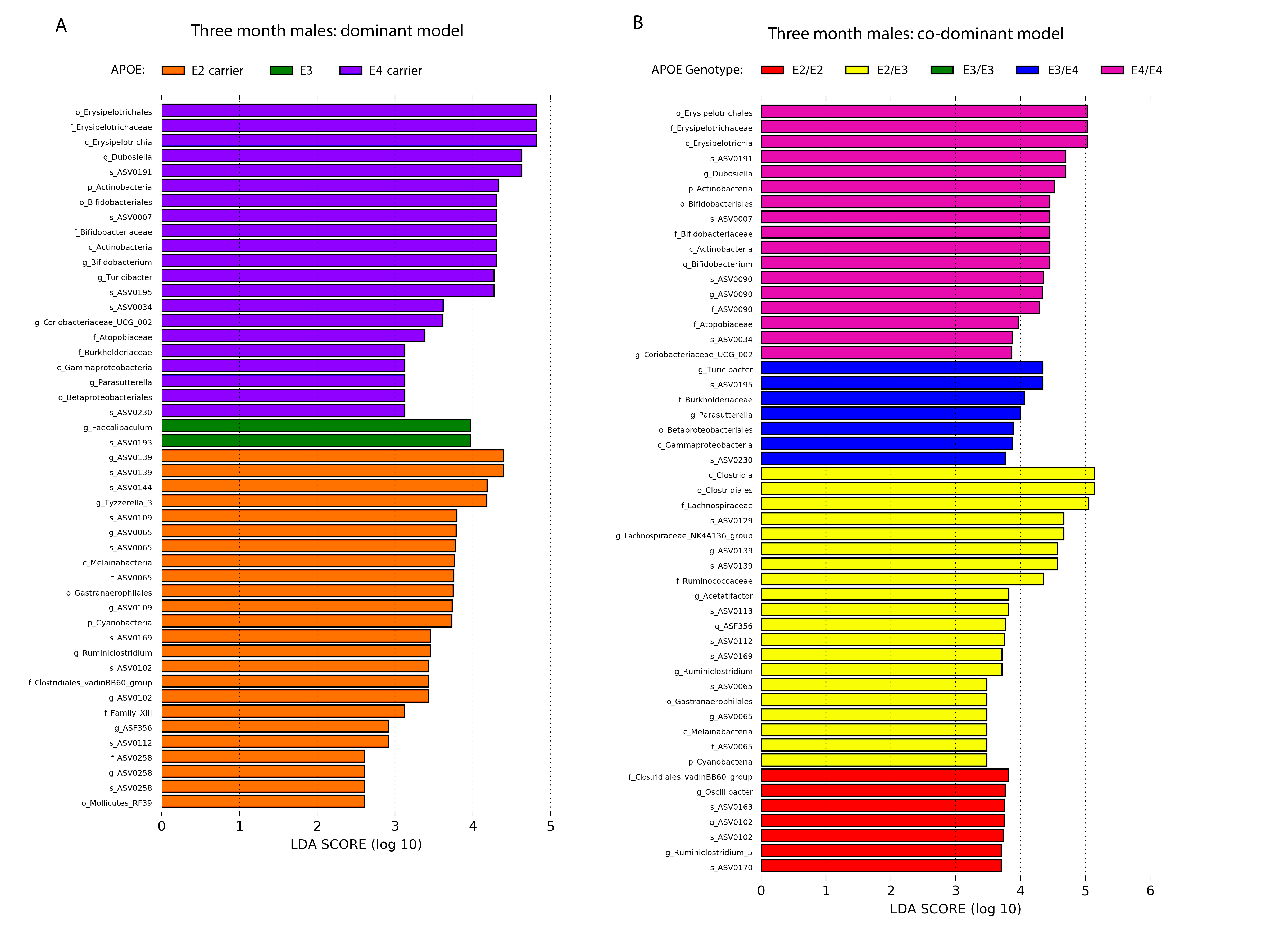

Supplement: Supplementary file 7 — Supplementary Figure S3. [file 41598_2022_5763_MOESM7_ESM.tif]
